# Ethanol Extract of *Brucea javanica* Seed Inhibit Triple-Negative Breast Cancer by Restraining Autophagy via PI3K/Akt/mTOR Pathway

**DOI:** 10.3389/fphar.2020.00606

**Published:** 2020-04-29

**Authors:** Xiaohong Chen, Shuang Li, Dan Li, Muxia Li, Ziren Su, Xiaoping Lai, Changlin Zhou, Shaodan Chen, Shunxian Li, Xiaobing Yang, Jiyan Su, Yunjian Zhang

**Affiliations:** ^1^State Key Laboratory of Applied Microbiology Southern China, Guangdong Provincial Key Laboratory of Microbial Culture Collection and Application, Guangdong Open Laboratory of Applied Microbiology, Guangdong Institute of Microbiology, Guangdong Academy of Sciences, Guangzhou, China; ^2^Guangdong Provincial Key Laboratory of New Drug Development and Research of Chinese Medicine, Mathematical Engineering Academy of Chinese Medicine, Guangzhou University of Chinese Medicine, Guangzhou, China; ^3^Department of Thyroid and Breast Surgery, the First Affiliated Hospital, Sun Yat-sen University, Guangzhou, China; ^4^Graduate School, Guangdong Medical University, Dongguan, China; ^5^Department of Research and Development, Guangdong Yuewei Edible Fungi Technology Co. Ltd., Guangzhou, China

**Keywords:** *Brucea javanica*, triple-negative breast cancer (TNBC), autophagy, PI3K/Akt/mTOR, toxicity

## Abstract

Triple-negative breast cancer (TNBC) is an aggressive disease with worst prognosis than other subtypes of breast cancer. Owing to the lack of hormone receptors and HER2 expression on TNBC cells, patients do not have targeted therapy options available with other breast cancer subtypes. Extensive efforts have been made to identify novel therapeutics against TNBC. Interestingly, recent studies had shown that plant-derived natural products could modulate the autophagy and induce the breast cancer cells death. Seed of *Brucea javanica* has been used as an important traditional Chinese medicine against cancers. In the present study, the anti-breast cancer potential of ethanol crude extracts from *B. javanica* seed (BJE) was explored. Data demonstrated that BJE could inhibit the TNBC cell line MDA-MB-231 proliferation and induced apoptosis. In the cells exposed to BJE, protein expressions of UNC-51-like kinase-1 (ULK1) and Beclin-1 and the ratio of light chain 3 II/I (LC3 II/I) were reduced, while the expression of p62 was increased, indicating an inhibition on autophagy. Moreover, BJE promoted the phosphorylation of mammalian target of rapamycin (mTOR), phosphatidylinositol 3-kinase (PI3K), and Akt in MDA-MB-231. BJE also suppressed the MDA-MB-231 tumor growth *in vivo*. Coincide with the results *in vitro*, autophagy in the tumor tissue was weakened as indicated by decreased ratio of LC 3 II/I and Beclin-1 accompanied by enhanced phosphorylation of mTOR, which confirmed that autophagy restraint via the PI3K/Akt/mTOR signaling pathway contributes to the suppression by BJE. Notably, no noticeable toxicity in non-targeted organs was found, including small intestine, liver, and kidney. Taken together, this study revealed anti-breast cancer activity of BJE based on autophagy restraint, highlighting its clinical importance as a novel natural agent against TNBC.

## Introduction

Breast cancer is one of the most commonly diagnosed cancers worldwide, and the leading cause of cancer-related deaths among females (Bray et al., 2018).Triple-negative breast cancer (TNBC) is an aggressive disease with worst prognosis than other subtypes of breast cancer. One of the major reasons is that TNBC cells do not have targeted therapy options available with other breast cancer subtypes, comparing to the hormone receptor positive and HER2-positive breast cancer. The recommended systemic treatment for TNBC is mainly concerned about chemotherapy, including anthracyclines, taxanes, anti-metabolic, alkylating, etc. (McDonald et al., 2016; Waks and Winer, 2019). Unfortunately, approximately one third of patients with early stage TNBC are still suffering from relapse or even died of breast cancer. Additionally, these common remedies are always accompanied by various side effects that cause systemic multi-organ damages, such as blood system, circulatory system, nervous system, digestive system, motor system, reproductive system, etc. Therefore, extensive efforts are being made to identify novel therapeutic agents to improve the prognosis of TNBC.

Autophagy is a self-protective biological process that maintains cellular homeostasis by balancing the biosynthetic and catabolic processes (Vessoni et al., 2013). Autophagy is a “double-edged sword” in all stages of cancer development. In the initial stage of cancer formation, the host itself would employ autophagy to reduce proteins and structural substrates for cell proliferation, so as to activate programmed cell death of damaged cells (Lin and Baehrecke, 2015). Hence, autophagy acts as a mechanism of tumor suppression at this stage. However in the developing stages, autophagy is the most optimal approach to endow cancer cells with metabolic flexibility, allowing for their survival in nutrient and oxygen-poor tumor microenvironments (TMEs). Extensive pre-clinical evidence suggests that autophagy restraint is benefit for the clinical outcomes in cancer patients. And chloroquine (CQ) and the related hydroxychloroquine (HCQ) are the most potential drugs that could be used to inhibit autophagy, especially in terms of sensitizing cancer cells to chemotherapy and radiotherapy (Sotelo et al., 2006). Interestingly, recent studies had shown that plant-derived natural products could modulate the autophagy and induce the breast cancer cells death (Wang et al., 2015; Tian et al., 2018). These studies implicated that strategies targeting autophagy have attracted increasing attention to develop novel remedies against breast cancer.

*Brucea javanica* (L.) Merr. (named Ya-dan-zi in Chinese) is a kind of shrubs that is widely distributed throughout southeastern Asia and northern Oceania (Dong et al., 2013). Seed of *B. javanica* (*Bruceae fructus*) has been used as an important traditional Chinese medicine against dysentery (Chinese Pharmacopoeia Commission, 2015) and inflammation (Yang et al., 2013). The oil of *B. javanica* has been developed in the form of injection and capsule, for the treatment of gastrointestinal cancer (Yan et al., 2015; Wu et al., 2018), encephalophyma, lung cancer and brain metastasis of lung cancer (Zhang et al., 2018). Moreover, compounds derived from *B. javanica*, such as a quassinoid named Bruceine D, exhibited pronounced anti-cancer activates in pancreatic cancer (Lai et al., 2017) and osteosarcoma (Wang et al., 2019). And these anti-cancer activities involves ROS regulation (Xie et al., 2019), phosphatidylinositol 3-kinase (PI3K)/Akt signaling pathway (Lai et al., 2017), JAK-STAT signaling pathway (Wang et al., 2019), and so on. In the present study, anti-cancer activity of the ethanol extract of *B. javanica* was explored from the perspective of autophagy to investigate its potential in the treatment of TNBC.

## Materials and Methods

### Animals

Female Balb/c nude mice (3 to 4-week old) were purchased from Guangdong Medical Laboratory Animal Center (Guangzhou, Guangdong, China). All animals were housed under the specific pathogen-free condition with controlled temperature (23 ± 2°C), humidity (50 % ± 5 %), and 12 h light/dark cycle, and were free access to food and water *ad libitum*. The *in vivo* experiment was performed after the 7-day acclimatization with the approval by Guangdong Institute of Microbiology Laboratory Animal Ethics Committee according to the guidelines (permission number: GT-IACUC201807262).

### Preparation and Analysis of Ethanol Extracts From *B. javanica* Seed (BJE)

The *B. javanica* seed was provided by Baiyunshan Mingxing Pharmaceutical Co., Ltd., and it was authenticated by Pro. Ziren Su (voucher specimen 20170121). The seeds of *B. javanica* were extracted with 95% ethanol at a ratio of 1:4 (weight/volume) by reflux extraction, and the procedure was repeated twice. The filtrates were pooled, concentrated under vacuum, and freeze-dried to yield BJE. BJE was stored 4°C prior to use.

High performance liquid chromatography (HPLC) analysis of BJE was carried out by liquid chromatography (Agilent Technologies 1200 Series). BJE was dissolved in methanol and separated on a Waters C18 column (250 mm × 4.6 mm, 5 μm) at 30°C. Water (A) and methanol (B) were used as mobile phase, and the following gradient program was set: 0–5 min, 5–5% B (v/v); 6–25 min, 10–45 % B (v/v); 26–40 min, 45–45% B (v/v); 41–55 min, 45–100% B (v/v); 56–60 min, 10–45% B (v/v). The sample was analyzed by Agilent UV detector at 240 nm.

### Cell Culture

Human TNBC MDA-MB-231 cell line was provided by Cell bank of Chinese Academy of Sciences, Shanghai, China. Cells were cultured in completed DMEM medium (4.5 mg/ml d-glucose, Gibco, NY) supplemented with 10 % fetal bovine serum (FBS, Gibco) and 1 % penicillin/streptomycin (Gibco), and maintained in incubators at 37°C under an atmosphere of 5% CO_2_.

### Cytotoxicity Test, Morphology Observation, and Apoptosis Assay

In all the cell experiments, BJE was dissolved in dimethylsulfoxide (DMSO) and then diluted with completed DMEM medium. The final concentration of DMSO was no more than 0.1%. For cytotoxicity test, cells were seeded in 96-well plates at a density of 3 × 10^3^ cells/ml (sextuple in each group), and treated with BJE (0.78 to 200 μg/ml) or PTX (7.8 to 2000 ng/ml). After 48 h, 3-(4,5-dimethyl-2-thiazolyl)-2,5-diphenyl-2-H-tetrazolium bromide (MTT, 5 mg/ml) was added to each well followed by 4 h incubation, and the optical density was measured at 490 nm by a Multiscan MK3 microplate reader (Thermo Fisher, USA).

For experiments except cytotoxicity test, cells were seeded in 6-well plates at a density of 1 × 10^5^ cells/ml (triplicate in each group), and treated with BJE (2.61, 5.21, 10.42 μg/ml) or PTX (26.04 ng/ml) for 48 h, and then the morphology of cell was captured by a light microscope (Olympus, Tokyo, Japan). For apoptosis assay, cells were harvested, washed with cold PBS, and stained with Annexin V (2.5 μL/test)/fluorescein isothiocyanate (FITC, 5 μL/test) (Lianke Biotech, Co., Ltd., 82480552) for 5 min at RT in the dark. Cell apoptosis was measured with a FACS Canto II cytometer (BD, USA), and the data was analyzed by Diva software (version 6.0).

### Xenograft Murine TNBC Model Induction and Treatment

MDA-MB-231 cells were subcutaneously (*s.c.*) injected into the right foreleg armpit of the Balb/c nude mice (0.1 ml/mouse, 2 × 10^6^ cells/mouse). After the tumor grew up to 1 mm^3^, the mice were randomly divided into six groups (6 mice in each group), including the model group, PTX group (Hannan Quanxing Pharmaceutical Co. Ltd., China), and the BJE groups. In the following 21 days, mice of the BJE groups were orally administrated with BJE (20 and 40 mg/kg) once a day, namely BJE-L (20 mg/kg) and BJE-H (40 mg/kg), respectively. Those of the PTX group were intraperitoneally (*i. p*.) injected with PTX (12.5 mg/kg) twice a week. The model group mice were given equal volume of distilled water.

Tumor volume was monitored with an electronic vernier caliper twice a week. The volume was calculated as V = a×b^2^/2, where a indicated the longer diameter, and b indicated the shorter diameter. On day 22, peripheral blood was collected from the orbital vein plexus. Then the mice were sacrificed by cervical dislocation to harvest tumor, small intestine, liver, and kidney. Tumors were weighed, photographed, and segmented. One part of the tumor, small intestine, liver, and kidney were fixed in 4% paraformaldehyde. Other parts of the tumor were snap-frozen in liquid nitrogen for either western blot, or kept in cold for real-time quantitative polymerase chain reaction (RT-qPCR).

### Hematoxylin-Eosin Staining

The fixed small intestine, liver, and kidney were embedded in paraffin, sliced into 3 μm thick sections, and subjected to hematoxylin-eosin (H&E) staining. The slides were observed under a light microscope (at 200 × magnification).

### Western Blot Analysis

For cells, protein was extracted from the cell lysate in RIPA buffer (Solarbio, Beijing, China) supplemented with protease inhibitor cocktail (Solarbio) after centrifuge (12,000 rpm for 30 min at 4°C). For the tumor, proteins were extracted by homogenization with Tissue total protein extraction kit (SolarBio Tech Co., Ltd., China). Protein concentration was determined by BCA protein assay kit and 20 μg proteins were separated by polyacrylamide gel-electrophoresis, and then transferred onto a PVDF membrane. Subsequently, the membrane was blocked on 5 % skim milk in TBST for 1 h. The primary antibodies against PI3K (Proteintech, Hubei, China), p-PI3K (anti-PI3 kinase p85 alpha (phospho Y607); Abcam, Cambridge, UK), mammalian target of rapamycin (mTOR; Cell Signaling Technology, MA, USA), p-mTOR (Cell Signaling Technology, Ser2448, MA, USA), ser/thr protein kinase (Akt, Cell Signaling Technology, MA, USA), p-Akt (Cell Signaling Technology, Ser473, MA, USA), LC3A/B (Cell Signaling Technology, MA, USA), Beclin-1 (Cell Signaling Technology, MA, USA), unc-51-like kinase 1 (ULK1, Cell Signaling Technology, MA, USA), p62/SQSTM1 (Cell Signaling Technology, MA, USA) and glyceraldehyde-3-phosphate dehydrogenase (GAPDH, Cell Signaling Technology, MA, USA) were then incubated overnight at 4°C. GAPDH was used as internal control to ascertain equal loading of proteins. Finally, the protein bands were detected with the enhanced chemiluminescence (ECL) detection reagents. The band intensity was quantified using Image J software (NIH Image, USA).

### Real-Time Quantitative Polymerase Chain Reaction Analysis

Total RNAs from tumor tissues were extracted with TRIzol according to the manufacturer’s instructions (Thermo Fisher Scientific, NY, USA). 3 μg of total RNA was reversed to cDNA with ReverAid First Strand cDNA Synthesis Kit (Thermo Scientific, MA, USA). Real-time Quantitative Polymerase Chain Reaction (RT-qPCR) reactions were performed with SYBR® Premix Ex TaqTM II (Takara Bio, Shiga, Japan) using Step One Plus Real-Time PCR system (Thermo Fisher Scientific, NY). The primer sequences were shown in **Table 1**.

**Table 1:** Primers for RT-qPCR.

Gene name	Primer		Product length (bp)
*LC3*	Sense	AACATGAGCGAGTTGGTCAAG	127
	Antisense	GCTCGTAGATGTCCGCGAT	
*AGT13*	Sense	TCCAGGCTCGGCTTGGTGAA	130
	Antisense	TGTCCTGCCAGTGCCTTCTTTG	
*AGT5*	Sense	TGGGCCATCAATCGGAAACTCA	129
	Antisense	TGCAGCCACAGGACGAAACAG	
*GAPDH*	Sense	TATGACAACAGCCTCAAGAT	104
	Antisense	AGTCCTTCCACGATACCA	

### Statistical Analysis

All data were expressed as mean ± standard deviation (SD). Statistical analysis was performed using Statistical Package for the Social Sciences (SPSS 22.0, Chicago, USA). The data were analyzed by one-way analysis of variance (ANOVA). Data was compared by post hoc LSD test under the condition of homogeneity of variance; if not, the Dunnett’s test was used. *p < 0.05 and **p < 0.01 *vs.* control group; #*p* < 0.05 and ##*p* < 0.01 *vs.* model group.

## Results

### BJE Inhibited MDA-MB-231 Cell Proliferation *In Vitro*

**Figure 1:**
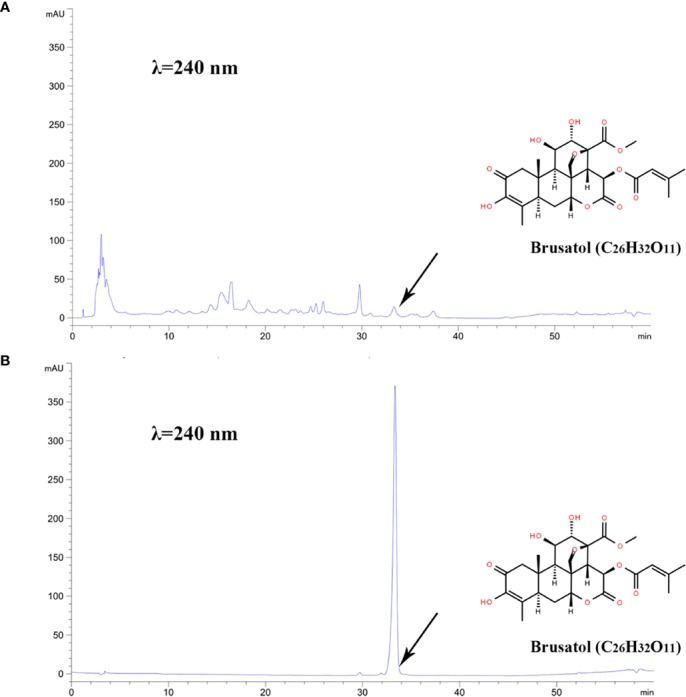
HPLC chromatogram. **(A)** BJE. **(B)** Brusatol, C_26_H_32_O_11_, retention time = 33.332 min.

**Figure 2:**
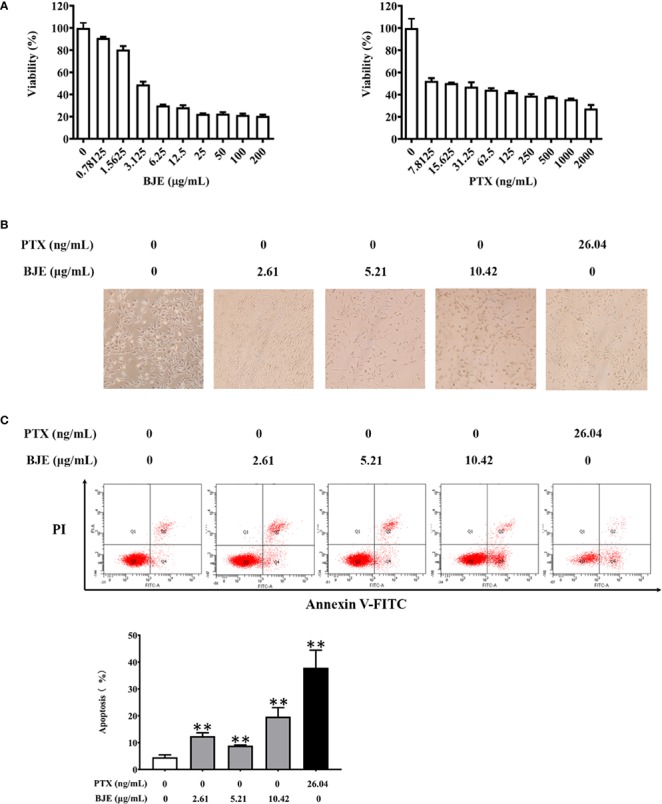
Cytotoxicity of BJE on MDA-MB-231. **(A)** Cell viability, n=6. **(B)** Cell morphology observation by light microscope. **(C)** Apoptosis by flowcytometry, n=3. Data was presented as mean ± SD. **p < 0.01 *vs* Control group.

The yield of BJE was 1.4%, and the chromatogram of BJE was showed in **Figure 1**. It was found that one of the reported compounds, brusatol, existed in BJE (**Figures 1A, B**). Cytotoxicity test by MTT assay showed that after the 48-h treatment, BJE significantly inhibited MDA-MB-231 cell proliferation in a dose-dependent manner (from 1.5625 to 200 μg/ml, 48-h treatment), and the half maximal inhibitory concentration (IC_50_) was 10.42 μg/ml, with 95% confidence interval (CI) ranging from 2.333 to 10.01 μg/ml (**Figure 2A**). PTX showed strong cytotoxicity in MDA-MB-231 cells with IC_50_ at 16.39 ng/ml with 95% CI ranging from 9.867 to 27.23 ng/ml. In terms of cell morphology (**Figure 2B**), cells treated with PTX (26.04 μg/ml) displayed evident nuclear condensation, while those treated with BJE (2.61, 5.21, and 10.42 μg/mL) showed distinct morphology, including shrinkage and vacuole formation. Moreover, BJE induced significant apoptosis in MDA-MB-231 (**Figure 2C**).

### BJE Inhibited Autophagy in MDA-MB-231 Cells by Activating the PI3K/Akt/mTOR Signaling Pathway

**Figure 3:**
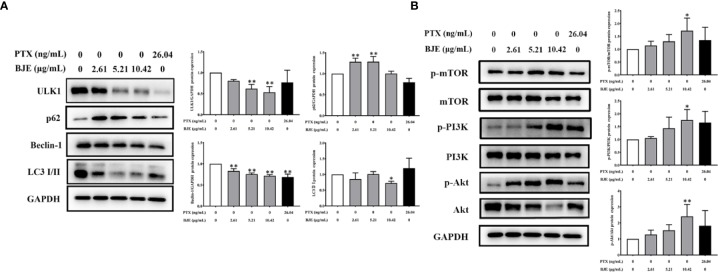
BJE inhibited autophagy in MDA-MB-231 cells. **(A)** Protein expressions of LC 3, p62, Beclin-1, and ULK1. **(B)** Phosphorylation of mTOR, Akt, and PI3K in MDA-MB-231 cells. n = 3, data was presented as mean ± SD. *p < 0.05 and **p < 0.01 *vs.* control group.

The possible involvement of autophagy was investigated to explore the underlying mechanism of the anti-proliferation activity by BJE. Data showed that BJE evidently suppressed the protein expression of ULK1 and Beclin-1, and the ratio of LC 3 II/I was also reduced with a remarkable increase of p62, indicating an autophagy inhibition in the BJE-treated MDA-MB-231 cells (**Figure 3A**). Moreover, phosphorylations of mTOR, PI3K, and Akt in MDA-MB-231 were significantly promoted by BJE (**Figure 3B**). These results suggested that the autophagy inhibition by BJE on MDA-MB-231 is closely related to the activation of PI3K/Akt/mTOR signaling pathway.

### BJE Suppressed MDA-MB-231 Tumor Growth Without Toxicity in the Non-Targeted Organs

**Figure 4:**
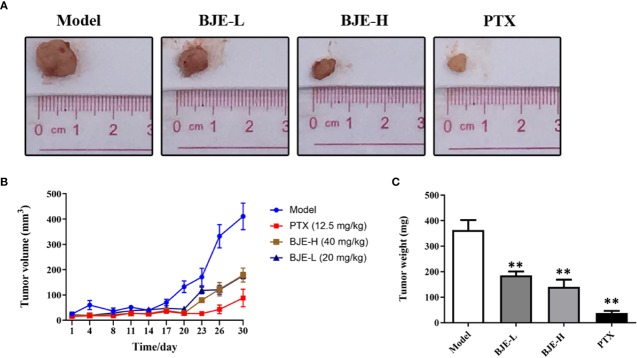
BJE suppressed MDA-MB-231 tumor growth *in vivo*. **(A)** Representative pictures of tumor. **(B)** Tumor weight. **(C)** Tumor volume. n=5–6, data was presented as mean ± SD. **p < 0.01 *vs.* Model group.

**Figure 5:**
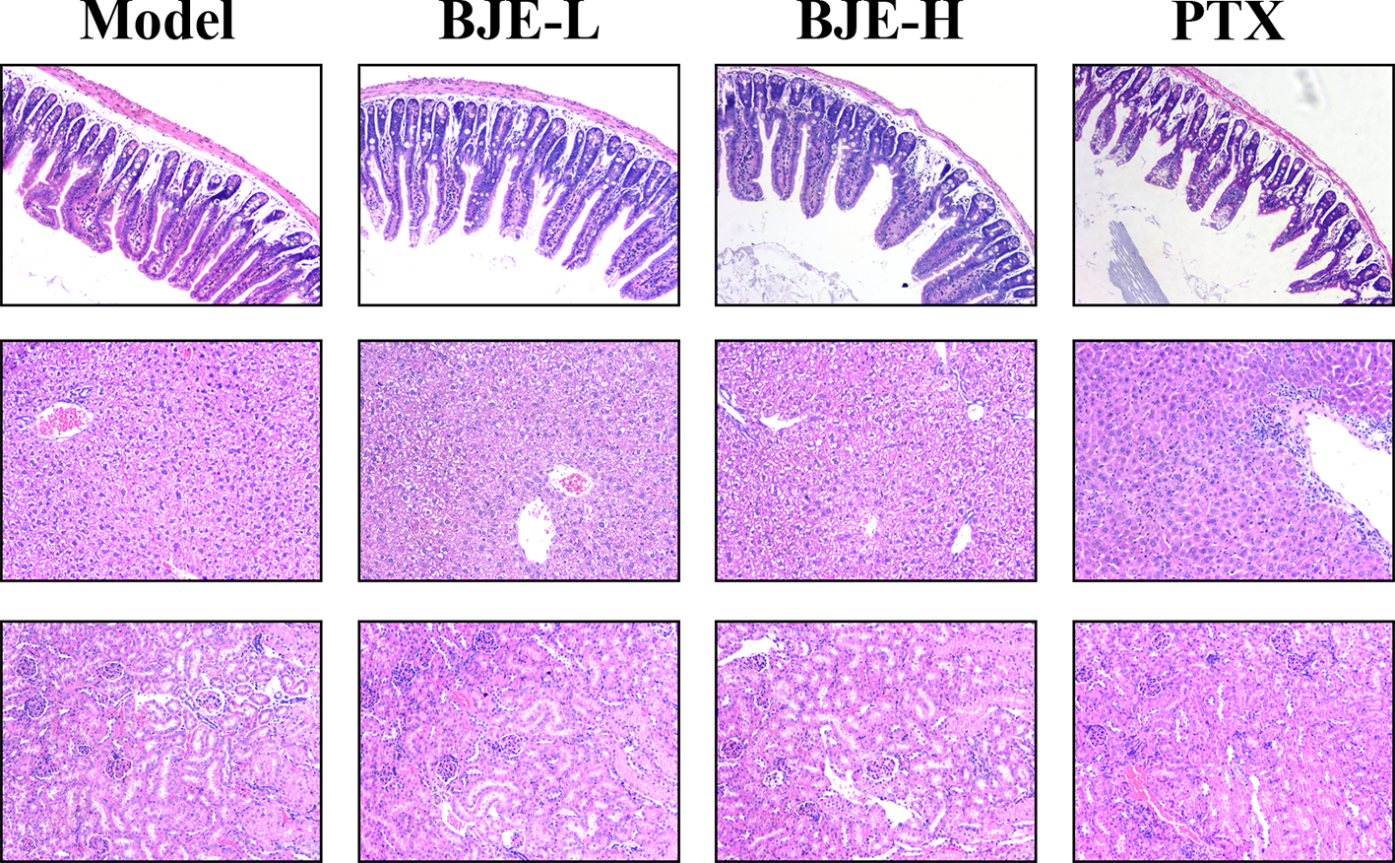
HE staining of the small intestine, liver and kidney (200×, n=5–6).

The MDA-MB-231-xenograft murine TNBC model was employed to confirm the tumor suppression effect of BJE. The MDA-MB-231 tumor growth was effectively suppressed by PTX and BJE during the 21-day treatment (**Figures 4A, B**). Finally, tumors of the PTX group was 38±20 mg; and those of the BJE groups were 186 ± 38 mg (BJE-L, 20 mg/kg) and 141±69 mg (BJE-H, 40 mg/kg), respectively, which were significantly lower than those of model group (364±96 mg, **Figure 4C**). H&E staining showed that although PTX effectively suppressed the tumor, it also caused obvious lesion in small intestine, which was featured by the shortened and atrophied villi, and the fractioned and incomplete muscular layer. PTX treatment also induced apparent inflammatory infiltration in liver, but it did not affect kidney. In contrast, BJE did not induced small intestine lesion or liver inflammatory infiltration in the tumor-bearing mice (**Figure 5**), implying that the anti-cancer activity of BJE was not accompanied by side effects in the non-targeted organs.

### Autophagy Restraint Contributed to the Suppression of MDA-MB-231 Tumor Growth by BJE

**Figure 6:**
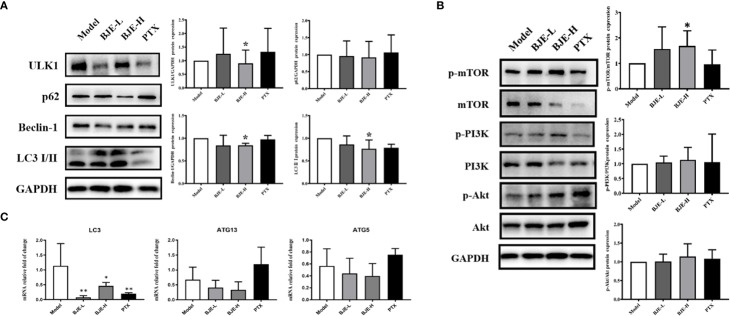
BJE restrained autophagy in the MDA-MB-231-xenograft murine breast cancer model. **(A)** Protein expressions of LC 3 II/I, p62, Beclin-1, and ULK1 in tumor tissue. **(B)** Phosphorylation of mTOR, Akt, and PI3K in tumor tissue. **(C)** mRNA levels of LC 3, ATG13 and ATG5 in tumor tissue. n=5–6, data was presented as mean ± SD. *p < 0.05 and **p < 0.01 *vs.* Model group.

As the *in vitro* studies indicated that BJE exhibited obvious inhibition on TNBC cells via autophagy restraint, autophagy status in tumor tissue was also examined. Protein expression of ULK1 and Beclin-1, and the ratio of LC 3 II/I were significantly reduced in the tumor tissue of BJE-H group (**Figure 6A**). In addition, the phosphorylated mTOR was increased in BJE-H group, demonstrating a suppression of autophagy by promoting the phosphorylation of mTOR (**Figure 6B**). Meanwhile, mRNA level of LC 3 was remarkably decreased in the tumor tissue of the BJE-H group, and a declining trend was also observed for ATG13 and ATG5 (**Figure 6C**). Together with the results of experiments *in vitro*, autophagy restraint via the activation of PI3K/Akt/mTOR signaling pathway would contribute to the suppression of TNBC cells by BJE.

## Discussion

Breast cancer, especially TNBC, remains a serious health challenge worldwide, and ongoing attempts have been made to explore and develop novel targets or treatment strategies for it. Although chemotherapy, endocrine therapy and targeting therapy agents have been used as clinical recommended systemic therapies, numerous breast cancer patients still suffered from relapses due to the tumor heterogeneity, moreover, side effects of these therapies have threatened quality of life and increased treatment cost (Jin and Ye, 2013; Tang et al., 2016). As alternative remedies, natural products have drawn increasing attention in cancer treatment due to their novel efficacies and safety. Seed of *B. javanica* has been reported as a promising source of natural products against various cancers (Lai et al., 2017; Wu et al., 2018; Zhang et al., 2018; Wang et al., 2019). In the present study, the ethanol extract of *B. javanica* seed (BJE) displayed evident inhibition on the TNBC cell line MDA-MB-231 *in vitro*, as well as the MDA-MB-231 tumor growth *in vivo*.

Necrosis, apoptosis, and autophagy are the three main cell death processes. It is generally recognized that necrosis is a passive form of non-programmed cell death that results from dramatic physical or chemical stimuli. Apoptosis and autophagy are active programmed cell death to eliminate abnormally proliferating cells for homeostasis maintenance, and they have been proposed as the targets in cancer therapies. In the proliferation inhibition by BJE, it was found that the cytotoxicity of BJE was dose-dependent, and the cells exposed to BJE displayed distinct shrunken and vacuolar morphology, which was different from the necrosis characteristics, such as swelling and cellular content leakage. On the other hand, flow cytometry showed that there existed apparent apoptosis in the cell death caused by BJE.

Autophagy acts as tumor suppressor or promoter depending on the stage of cancer development. Generally, autophagy is maintained at a basal level in all cells under the control of highly regulated set of signaling events, which mainly involves evolutionarily conserved genes called autophagy-related genes (*ATG*) (Towers and Thorburn, 2016). Autophagy is triggered by diverse signals (such as the PI3K/Akt/mTOR signaling pathway) and cellular stress (nutrient deprivation, hypoxia or metabolic stress), although the distinction between basal and stimulated autophagy is poorly understood. During the advanced stages, autophagy is positively enhanced and promotes tumor cell proliferation by absorbing nutrients and energy (White et al., 2015), which would also suppress apoptosis. The initiation of autophagy begins with the activation of the ULK1 (also known as ATG1) complex with ULK1, ULK2, ATG13, and FIP200. The ULK1 complex up-regulates Beclin-1 and promotes the formation of class III PI3K complex that contains vacuolar protein sorting-34 (VPS34, also known as PIK3C3), p150, Beclin-1, and ATG14 or UV radiation resistance-associated gene protein (UVRAG; also known as p63) (Liang et al., 1999). Then the autophagosome membrane expands by the conjugation of ATG5-ATG12 complex with ATG16. Meanwhile, LC3-I is conjugated with lipid phosphatidylethanolamine (PE) by the conjugation of ATG4B and ATG7 to form LC3-II, which is finally recruited to the membrane. Hence, this lipid-conjugated form of LC3 is well established as an autophagosome marker (Klionsky et al., 2016). Ultimately, contents of the autophagosome are degraded as macromolecular precursors that are recycled or used to fuel metabolic pathways after the autophagosome has fused with the lysosome. During this process, autophagic flux can be measured by the degradation of the adaptor protein sequestosome 1 (also known as p62), which is degraded along with other cargo proteins that are critical substrates to autophagosomes and LC3II (Klionsky et al., 2016). The present study showed that BJE down-regulated the protein expressions of ULK1, Becline-1, and reduced the lipidation of LC 3 (ratio of LC 3 II/I) with increased p62 in MDA-MB-231 *in vitro*, and reductions of LC 3 lipidation, ULK1, and Beclin-1 were found in the tumors of BJE-treated mice. These changes of autophagy-related proteins indicated an autophagy restraint induced by BJE. Recent studies demonstrated that compared with the other types of breast cancer, basal autophagy of TNBC, such as the MDA-MB-231 cell line, was higher due to the substantially higher number of autophagosomes (Maycotte et al., 2014; Garbar et al., 2017). Similar to a previous study (Garbar et al., 2017), the expression of LC3b (or the ratio of LC 3 II/I) was comparable in the MDA-MB-231 cells of the control group and the PTX group, while BJE inhibited this event for autophagy. These results suggested that tumor suppression of BJE could be possibly attributed to an autophagy restraint.

The PI3K/Akt/mTOR signaling pathway is a well-recognized upstream of autophagy. As demonstrated in yeast (Noda and Ohsumi, 1998), drosophila (Scott et al., 2004), and mammalian cells (Kim et al., 2011), mTOR exerts its crucial effect on autophagy as a downstream component in the signaling pathway of PI3K/Akt. Under normal conditions, phosphorylated PI3K phosphorylates Akt, which inhibits tuberous sclerosis complex 1/2 (TSC1/2) and then activates mTOR (Kim and Guan, 2015). Subsequently, mTOR negatively regulates autophagy via suppressing ULK1 that coordinates the autophagy initiation (Jung et al., 2009). Two mechanisms are employed by mTOR to inhibit ULK1. First, mTORC directly phosphorylates ULK1 Ser 757 and disrupts the interaction between ULK1 and AMP-activated protein kinase (AMPK), through association of the mTORC1 component, Raptor, with ULK1 (Hosokawa et al., 2009; Kim et al., 2011). Second, mTORC indirectly destabilizes ULK1 through phosphorylation of Autophagy/Beclin-1 regulator 1 (AMBRA1), which impairs the ubiquitylation of ULK1, as well as the following stabilization, self-association and function (Nazio et al., 2013). The present study showed that as autophagy was inhibited, MDA-MB-231 cells exposed to BJE displayed higher phosphorylation of mTOR, PI3K, and Akt *in vitro*, and the MDA-MB-231 tumor tissue from BJE groups exhibited obviously higher phosphorylation of mTOR, implying that BJE is able to suppress autophagy via activating the PI3K/Akt/mTOR signaling pathway, thus blocking the development of TNBC.

Notably, the tumor suppression of BJE was not accompanied by side effects. Chemotherapy is known to cause various side effects, such as peripheral neuropathy, nephrotoxicity, myelotoxicity, hypersensitivity, and mucositis. Mucositis is a common side effect, which results in dyspepsia, dysphagia, malabsorption or diarrhea (Fu et al., 2018). The results showed that toxicity in small intestine, liver, and kidney, was rarely found after oral administration of BJE, indicating a clinical potential of BJE due to its efficacy in tumor suppression as well as its safety in the non-targeting organs.

## Conclusion

This study revealed an anti-TNBC potential of ethanol crude extracts from *B. javanica* seed (BJE). BJE inhibited the TNBC cell line MDA-MB231 proliferation, whereby autophagy was weakened by activation of the PI3K/Akt/mTOR signaling pathway. Tumor suppression *in vivo* also confirmed that autophagy restraint via the PI3K/Akt/mTOR signaling pathway contributed to the anti-cancer activity of BJE, thereby highlighting its clinical importance as a novel natural agent against TNBC.

## Data Availability Statement

All datasets generated for this study are included in the article/supplementary material.

## Ethics Statement

The animal study was reviewed and approved by the Institutional Animal Care and Use Committee of Guangdong Provincial Hospital of Chinese Medicine.

## Author Contributions

JS and YZ conceived and designed the experiments. XC, SaL, DL, ML, ZS, XL, CZ, SC, SxL, and XY carried out the experiments. XC and SaL drafted the manuscript and JS and YZ revised the manuscript. All authors have reviewed and approved the final version of the manuscript.

## Funding

This work was financially supported by National Natural Science Foundation of China (81902709, 81872344), Natural Science Foundation of Guangdong Province (2018A0303130102, 2018A030313887), Guangdong Province Innovation Team Construction Program on Modern Agriculture Industrial technology system (2019KJ103), Science and Technology Planning Project of Guangdong Province (2017A050506044), Science and Technology Planning Project of Guangzhou City (201704030028), Key Program for Subject Research of Guangzhou University of Chinese Medicine (XK2019002), GDAS’ Special Project of Science and Technology Development (2019GDASYL-0105002), and High-level Leading Talent Introduction Program of GDAS (2016GDASRC-0102).

## Conflict of Interest

Author SxL was employed by the company Guangdong Yuewei Edible Fungi Technology Co. Ltd.

The remaining authors declare that the research was conducted in the absence of any commercial or financial relationships that could be construed as a potential conflict of interest.
